# Lung Ultrasound in Pediatrics: A Review with Core Principles That Every User Should Know

**DOI:** 10.3390/diagnostics15212782

**Published:** 2025-11-02

**Authors:** Soultana Foutzitzi, Panos Prassopoulos, Athanasios Chatzimichail, Katerina Kambouri, Hippocrates Moschouris, Evlampia A. Psatha, Panagoula Oikonomou, Savas P. Deftereos

**Affiliations:** 1 Department of Radiology, Alexandroupolis University Hospital, Democritus University of Thrace, 68100 Alexandroupolis, Greece; hipmosch@gmail.com (H.M.); eviepsatha@yahoo.gr (E.A.P.); sdefter@med.duth.gr (S.P.D.); 2 Department of Clinical Radiology, AHEPA University Hospital of Thessaloniki, Aristotle University of Thessaloniki, 54636 Thessaloniki, Greece; pprasopo@auth.gr; 3 Department of Pediatrics, Democritus University of Thrace, 68100 Alexandroupolis, Greece; 4 Department of Pediatric Surgery, Alexandroupolis University Hospital, Democritus University of Thrace, 68100 Alexandroupolis, Greece; kampkat@gmail.com; 5 Second Department of Surgery, Faculty of Medicine, Alexandroupolis University Hospital, Democritus University of Thrace, 68100 Alexandroupolis, Greece; paoikono@med.duth.gr

**Keywords:** lung ultrasound, respiratory distress syndrome, interstitial pathologies, pediatrics, neonatal

## Abstract

Lung ultrasound (LUS) has emerged as a valuable diagnostic modality for the evaluation of respiratory disorders in neonates, infants and children. LUS has high diagnostic accuracy for identification of lung lesions in neonates, infants and children, where most lung lesions abut the pleura. Furthermore, LUS has the advantage of rapid execution and ease of use, and does not require ionizing radiation. Its sensitivity, cost-effectiveness, and clinical efficiency make it an important tool for supporting clinical decision-making and improving patient management. Moreover, LUS may represent a reliable alternative to chest radiography for the assessment of pediatric lung conditions and, in selected cases, could potentially replace routine chest X-rays (CXRs). Because LUS is a user-friendly technique that enables real-time imaging without radiation, it has increasingly been used in clinical practice in recent years. Here, we discuss the diagnostic role of LUS for the accurate identification of pulmonary lesions in pediatric patients. In addition, we present LUS sonographic findings associated with common pediatric lung diseases, including signs and artifacts that can be used during diagnosis and evaluation of pediatric patients.

## 1. Introduction

Chest X-rays are considered to be the most useful and valuable imaging modality for the diagnosis of lung diseases. However, X-rays cause radiation damage to patients, and neonates, infants and children are more susceptible to radiation damage because they have rapidly dividing cells that are less efficient at repairing mutated DNA [1]. Thus, LUS has been increasingly used in clinical practice as an alternative imaging application. Numerous studies have shown that LUS is a “baby-friendly” method that can provide accurate and reliable diagnosis and allow for the follow-up monitoring of lung diseases, especially in the context of neonatal patients [2,3].

The spectrum of lung diseases includes several heterogeneous conditions with diffuse interstitial involvement, which can result in the impairment of alveolocapillary exchange capacity and lead to severe respiratory failure. In this manuscript, we present the current state of knowledge of LUS and provide a comprehensive description of the principal diagnostic findings and signs observed in LUS in the context of specific pediatric pulmonary pathologies. Throughout this review and in associated images, an attempt has been made to describe how to differentiate normal lungs from pathologic conditions using specific artifacts, such as A-lines vs. B-lines, the presence or absence of “lung sliding”, the “lung point”, etc. [3,4,5].

## 2. Relevant Sections

As LUS is increasingly becoming part of the daily diagnostic approach in children—and especially in newborns—there is a growing need for familiarization with the method, as well as a deeper understanding of its principles and applications. Furthermore, it is important to recognize that, in LUS, we do not image anatomical structures (such as the lungs) directly, but instead evaluate artifacts generated by ultrasound. Therefore, as can be easily understood, expensive equipment is not required, and the use of specialized software is actually discouraged. In this review, we present the key artifacts observed in LUS, which may be considered “technical errors” encountered during the evaluation of pulmonary pathology in neonates, and more broadly, in the pediatric population. These include the following:

1. A-lines;
2. B-lines;
3. Pleural line;
4. Sea-shore sign;
5. Sinusoid sign;
6. Lung point sign;
7. Double-lung-point sign;
8. White lung.

Following this, we describe the most common pathologies observed in the neonatal age group, based on their sonographic appearance, clinical significance, and frequency.

## 3. Discussion

One of the major advantages of LUS is portability: because the ultrasound device is relatively small and light and only requires a convex and linear probe, it can be brought directly to the patient’s bedside, eliminating the need to transport pediatric patients to the radiology department. Bedside application of LUS, combined with a thorough medical history and clinical examination, has the potential to reduce the need for chest radiographs and improve the management and treatment of pediatric patients with respiratory diseases. Because of this, interest in the use of LUS for the diagnosis and monitoring of pediatric patients has been steadily increasing.

LUS findings, such as subpleural consolidations less than 1 cm in diameter, as well as the presence of distinct or coalescent B-Lines and other signs, are frequently observed in pediatric patients with respiratory illnesses. However, in the case of discrepancies between LUS findings and clinical or laboratory data, a CXR or, in more complex cases, a computed tomography (CT) scan should also be performed [6,7].

To make LUS more operator-friendly, there is a clear need to establish standardized training protocols, aimed at improving the interpretation and evaluation of LUS imaging findings. In parallel, broader investigation of the capabilities of LUS for the evaluation of pediatric and neonatal patients could help facilitate clinical diagnosis in children with suspected respiratory pathologies, especially in cases where the diagnosis is uncertain or unclear [8].

However, LUS is also associated with several known disadvantages, and the commonly reported ones among them are listed below:

1. LUS is an operator-dependent method.
2. LUS has a significantly limited ability to image paravertebral areas, particularly those below the angle of the scapula.
3. The quality and accuracy of LUS imaging can be negatively affected by obesity and/or the presence of subcutaneous emphysema.
4. Identification of air leak syndromes such as pneumomediastinum, pneumopericardium and interstitial emphysema, as well as air trapping and sub-alveolarization, are difficult using only LUS imaging methods [6,9,10,11].

### 3.1. Key Artifacts and Diagnostic Signs in Lung Ultrasound Imaging: A Comparative Overview of Normal and Pathological Findings

#### 3.1.1. A-Lines

**Figure 1:**
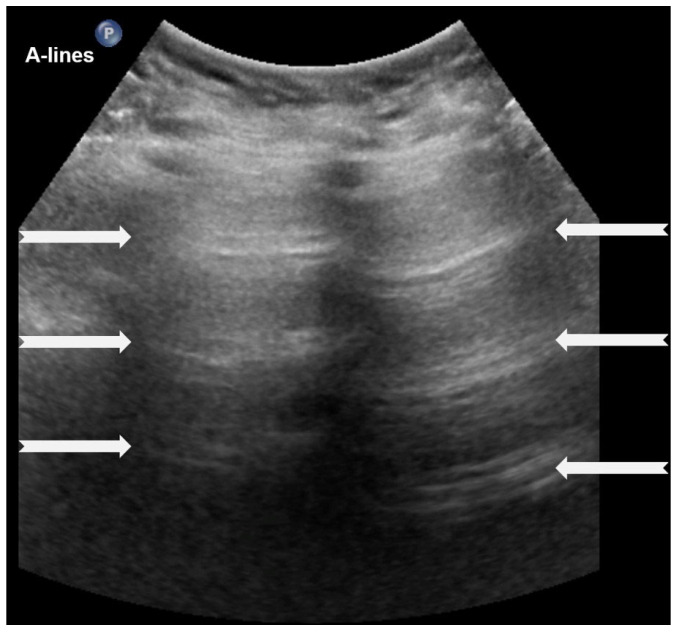
A-lines: Horizontal, bright, echogenic lines at equal intervals below the pleural line, running parallel to it. A-Lines are one of the main (basic) artifacts in LUS.

A-lines are horizontal, bright, echogenic lines that appear at equal intervals below the pleural line, running parallel to it (Figure 1). They are reverberation artifacts caused by the reflection of ultrasound waves between the pleura and the ultrasound probe. Although their presence typically indicates well-aerated, healthy lung tissue, A-lines can also be seen in certain pathological conditions, such as pneumothorax, where free air is present. Thus, A-lines are not always necessarily a sign of normal, healthy lungs [5,6,12,13,14,15,16].

#### 3.1.2. B-Lines

**Figure 2:**
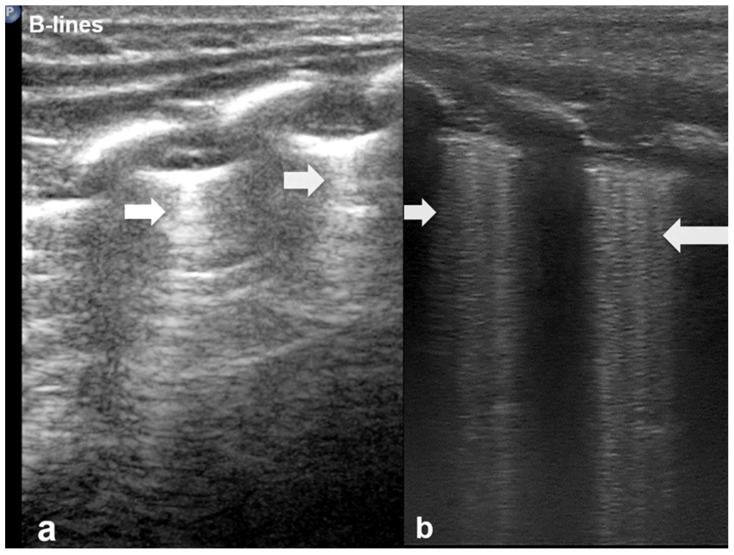
B-lines: Vertical reverberation-like hyperechoic artifacts originate from the pleural line and extend to the bottom of the image (**a**). This artifact could be normal (<3 in an intercostal space) or abnormal when are more than three, multiple or coalescent B-lines (**b**).

B-lines are vertical reverberation-like hyperechoic artifacts seen during LUS imaging which originate from the pleural line and extend to the bottom of the imaging field without fading in intensity (Figure 2a). B-line artifacts move in synchrony with lung sliding and intersect horizontal A-lines, effectively obscuring them. Their presence is typically associated with fluid accumulation in the alveoli or interstitial space, and they are commonly referred to as ultrasound lung comets. However, B-lines are frequently observed in neonates and can appear as a normal finding within the first 24 to 48 h after birth [13,14,17]. During LUS imaging, identification of up to two B-lines at the lung base is considered to be normal, while the presence of three or more B-lines between the intercostal space in a single view is considered significant and indicative of an underlying pathology. If multiple B-lines are identified by LUS, then the patient likely suffers from interstitial syndrome. When the air content in the lungs decreases, the number of lines tends to increase, because of the associated increase in lung density. Moreover, when the identified B-lines are confluent, this is an indication that the alveoli are filled with sub-pleural fluid. When interstitial edema is present, the presence or absence of B-lines can guide the administration of fluids [16,17,18,19]. However, it should be noted that B-lines are absent in the aforementioned pneumothorax.

**Figure 3:**
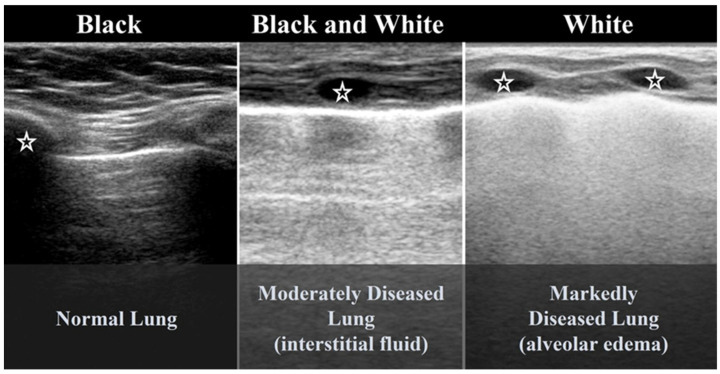
Black, black and white, white LUS patterns. (Stars: ribs).

According to severity of the lung or interstitial pathology, B-lines may be solitary, few, multiple or even coalescent (Figure 2b), resulting in three imaging patterns associated with increasing severity: black lung, black and white lung, or white lung (Figure 3) [20,21].

#### 3.1.3. Pleural Line

**Figure 4:**
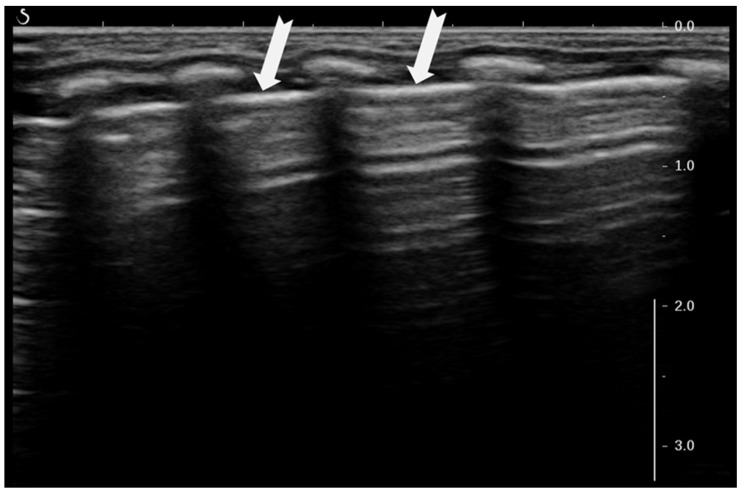
The pleural line, the only lung structure clearly visible with LUS; it must be homogeneous and hyperechoic.

The pleural line (Figure 4) appears as a bright, horizontal echo on LUS images and marks the boundary where tissue from the chest wall contacts the lungs. This echogenic line results from the significant difference in acoustic impedance between the soft tissues of the chest wall, such as muscle and subcutaneous fat, which are fluid-filled, and the air-filled lungs. It represents the interface between the parietal and visceral pleura [4,5,12,17,20].

#### 3.1.4. Sliding Lung Sign

**Figure 5:**
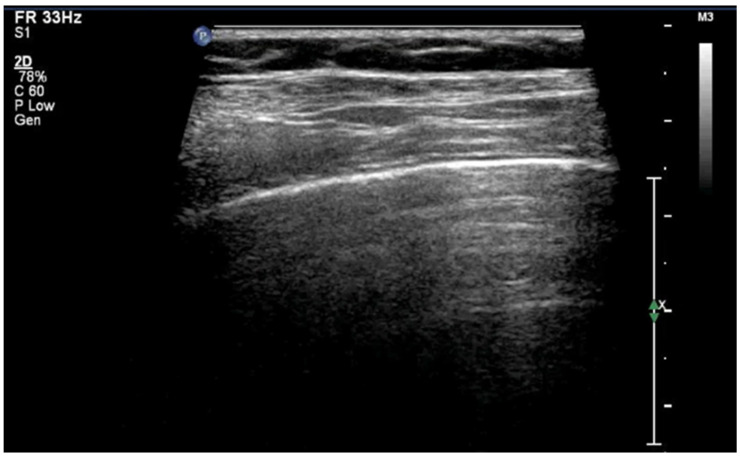
Sliding lung sign (a real-time image or video is needed to understand the movement).

The “sliding lung sign” is a key dynamic ultrasound finding that indicates normal lung expansion and pleural integrity. It refers to the respiratory-synchronous movement of the pleural line, which represents the interface between the parietal pleura lining of the chest wall and the visceral pleura covering the lung surface, which appears as a shimmering or “ants on a log” motion in ultrasound images. This shimmering or “ants on a log” motion seen during real-time ultrasound is caused by the relative displacement of the pleural layers during ventilation, as the lung expands and contracts against the inner thoracic cavity (Figure 5) [6,15].

The presence of the “sliding lung sign” confirms normal pleural apposition and effectively rules out pneumothorax at the site of examination. ‘Sliding lung’ is best visualized in B-mode and confirmed using M (motion)-mode, where it is associated with a characteristic “seashore sign” (see definition below). The presence of these signs further supports normal lung movement, while their absence can suggest pleural separation, as seen in pneumothorax, pleural adhesions, some cases of severe lung consolidation, or even tumors. Clinically, the sliding lung sign serves as a critical marker of pleural contact and is essential for the bedside LUS assessment of neonates, infants, children and adults [15].

#### 3.1.5. Sea-Shore Sign

**Figure 6:**
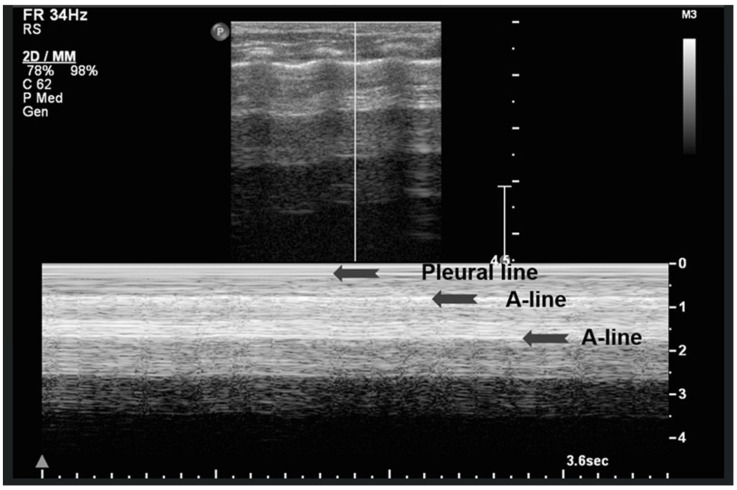
Upper image: LUS with normal A-Lines in B (bright)-mode. The simultaneous M (motion)-mode image (lower image) reveals series of horizontal echogenic lines which represent the pleural line and its reverberations (A-lines), as well as the air-filled alveoli with a granular/sandy appearance (more prominent in real time scan). These in combination produce the seashore sign.

The “sea-shore sign” is a characteristic ultrasound finding observed during M-mode (motion mode) scanning, and is indicative of normal lung sliding. In this pattern, the pleural line and overlying chest wall structures produce a series of horizontal echogenic lines, representing static tissues (Figure 6).

In contrast, the underlying lung parenchyma, which moves in synch with respiration, generates a granular or sandy appearance due to the dynamic motion of air-filled alveoli [17]. This combination creates the visual effect of waves (static layers) above and sand (with a dynamic speckled texture) below, thus producing the so-called “sea-shore sign”. The presence of this sign confirms normal apposition and movement of the visceral and parietal pleurae, and can be used to effectively rule out pneumothorax at the examination site. The “sea-shore sign” is considered to be a reliable and non-invasive indicator of intact lung sliding, and is frequently used in both adult and neonatal lung ultrasound to assess respiratory function and pleural integrity [5,6,15,22,23].

When this sign is absent, uniform horizontal straight lines, known as the stratosphere sign or “barcode sign”, appear, indicating pneumothorax as a possible cause [18,24].

#### 3.1.6. Sinusoid Sign

**Figure 7:**
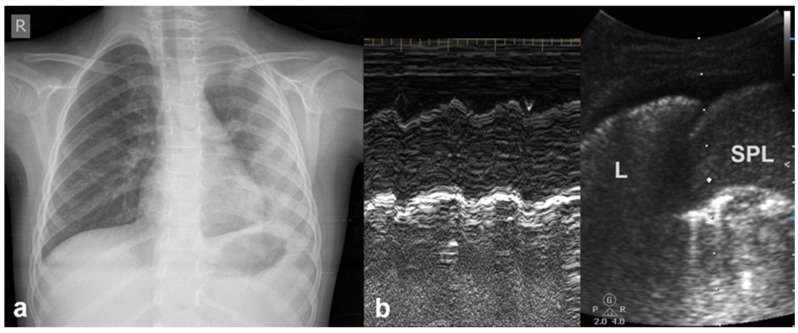
Sinusoid sign: (**a**) CXR: left-sided consolidation (is there any pleural effusion?). (**b**) LUS reveals fluid, a finding based on the sinusoid sign, with a characteristic sinusoidal pattern due to the cyclical movement of the lung line toward the pleural line during inhalation and away from the pleural line during exhalation (L: lung; SPL: spleen).

In cases of pleural effusion, the accumulation of fluid between the pleural layers causes separation of the visceral and parietal pleura. LUS assessment of this condition in M-mode is associated with the presence of a characteristic sinusoidal pattern, resulting from the cyclical movement of the lung line toward the pleural line during inhalation and away from the pleural line during exhalation. The presence of this “sinusoid sign” indicates the presence of low-viscosity fluid within the pleural space, separating the visceral and parietal pleura. This finding can assist in differentiating pleural effusion from pleural thickening, as its presence is indicative of mobile, free-flowing fluid typically associated with pleural effusions (Figure 7) [17].

#### 3.1.7. Lung Point Sign

**Figure 8:**
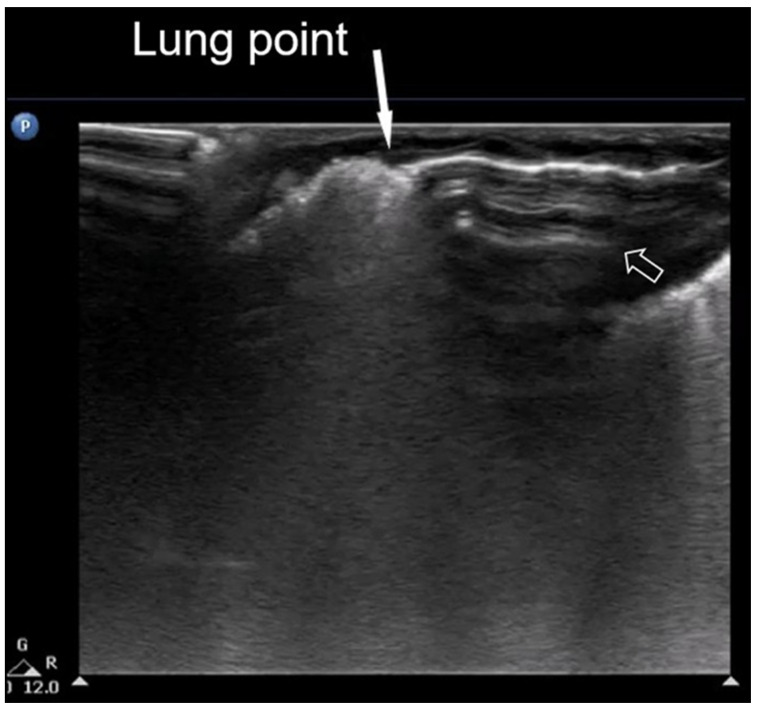
Lung point sign: The transition point between the aerated lung with coalescent B-lines (which probably represents respiratory distress syndrome) which are absent in pneumothorax and the area with A-lines (transparent arrow) but without lung sliding (absent in pneumothorax in real-time scanning).

The “lung point” or “lung point sign” is a highly specific lung sonographic sign which helps in the diagnosis of pneumothorax and can also facilitate the assessment of pneumothorax severity. In particular, the severity of pneumothorax depends on its position relative to the anatomical lines that define the regions of the chest. In LUS, the “lung point” is defined as the boundary between the area without normal lung sliding (i.e., pneumothorax) and the area where normal lung sliding is present (normal lung) (Figure 8) [4,12,15,16,25,26].

#### 3.1.8. Double-Lung-Point Sign

**Figure 9:**
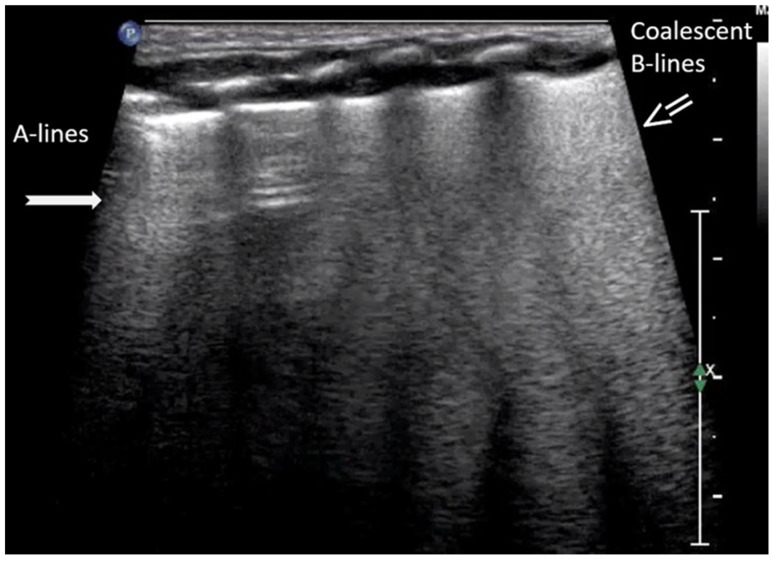
Double-lung-point sign: Normally aerated upper lung fields (arrow) and lower lung zones with multiple confluent B-lines (open arrow).

The “double-lung-point sign” is a sharp sonographic demarcation visible between normally aerated upper lung fields and lower lung zones, with multiple and/or confluent B-lines indicative of interstitial edema (Figure 9).

This pattern is characteristic of Transient Tachypnoea of the Newborn (TTN) [27,28], where a marked difference in lung echogenicity between superior and inferior regions can be visualized by ultrasound [6,26]. This finding has been reported to have a specificity of up to 100% for TTN [6,17,26], and is not typically present in other neonatal pulmonary conditions such as atelectasis, pneumothorax, pneumonia, or pulmonary hemorrhage [17,29].

#### 3.1.9. White Lung

LUS is a very useful tool for the follow-up monitoring of neonates. Specifically, as interstitial and alveolar fluids are reabsorbed, B-lines gradually disappear; however, B-lines may persist for much longer in neonates with lower gestational age and in those delivered by cesarean section. When B-lines become confluent, producing a “white lung” pattern, they are typically observed due to the presence of a relatively higher fluid content and a lower glomerular filtration rate [8].

**Figure 10:**
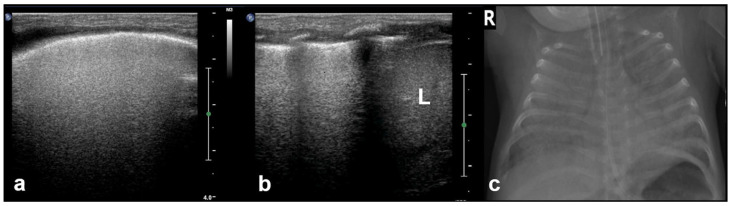
White lung: (**a**) axial plane, (**b**) longitudinal plane (L: liver), and (**c**) CXR.

The term “white lung” with respect to neonatal lung ultrasound refers to a sonographic pattern in which the lung fields are entirely filled with compact B-lines in every examined intercostal space, resulting in a uniformly hyperechoic appearance across the lung surface. The compact B-lines are so densely packed that they obliterate A-lines and give the lung a bright, homogenous appearance on ultrasound—hence the term “white lung” (Figure 10).

This white appearance during LUS imaging can be caused by a range of problems, including fluid buildup in the air spaces and interstitial space of the lungs, inflammation of the lung tissue, and scarring of the lung tissue. This artifact and pattern are typically associated with widespread pulmonary edema or alveolar–interstitial syndrome and may be seen in conditions such as respiratory distress syndrome (RDS) or Transient Tachypnoea of the Newborn (TTN) [19,30,31,32].

Importantly, white lung differs from other lung ultrasound findings such as localized consolidations or spared areas. It reflects diffuse interstitial–alveolar syndrome and is generally not present in milder pathologies or localized respiratory disease. Recognition of the white lung pattern in LUS is crucial for early diagnosis and severity assessment, particularly in neonates presenting with generalized respiratory distress [30].

### 3.2. Main LUS Findings Associated with Neonatal Lung Diseases

#### 3.2.1. Alveolar–Interstitial Syndrome

Alveolar–interstitial syndrome (AIS) can be either acute or chronic, and encompasses a range of pulmonary conditions characterized by the pathological involvement of both the alveolar and interstitial compartments of the lung. In neonates, AIS is commonly observed in conditions such as transient TTN, RDS, and early-stage bronchopulmonary dysplasia (BPD), whereas in adults, pulmonary fibrosis, acute respiratory distress syndrome (ARDS), acute pulmonary edema, and interstitial pneumonia are possible [12,17,33].

Neonatal respiratory diseases pose a diagnostic dilemma for radiologists, because chest X-rays cannot always provide proper diagnosis due to observer variability. Accurate diagnosis requires a significant level of knowledge, experience and careful observation of chest X-rays by the physician [6]. Thus, use of LUS in neonatal patients as a first-line imaging technique is strongly recommended, due to its ability to enable rapid, dynamic monitoring of changes in the pulmonary status of neonates. However, it should be noted that LUS is also often falsely considered to be an easy method to apply.

LUS is becoming a useful tool in neonatal intensive care units for the differential diagnoses of several pathologies, including RDS, Transient Tachypnea of the Newborn, neonatal pneumonia, pulmonary hemorrhage, meconium aspiration syndrome, pneumothorax and bronchopulmonary dysplasia [16,34].

#### 3.2.2. Transient Tachypnoea of the Newborn—Respiratory Distress Syndrome

RDS and TTN are common pathological entities that share similar clinical features, but present with distinct lung ultrasound patterns. RDS is caused by a deficiency or inactivation of pulmonary surfactants, whereas TTN results from the delayed or inadequate clearance of fetal alveolar fluid. LUS serves as a valuable adjunctive diagnostic tool, with a high level of accuracy, sensitivity and specificity for the diagnosis and differentiation of RDS and TTN [34].

#### 3.2.3. Transient Tachypnoea of the Newborn (TTN)

In TTN, LUS findings include the double-lung-point sign, pulmonary edema, alveolar–interstitial syndrome, compact B-lines, and a regular pleural line without subpleural consolidation [20,35].

**Figure 11:**
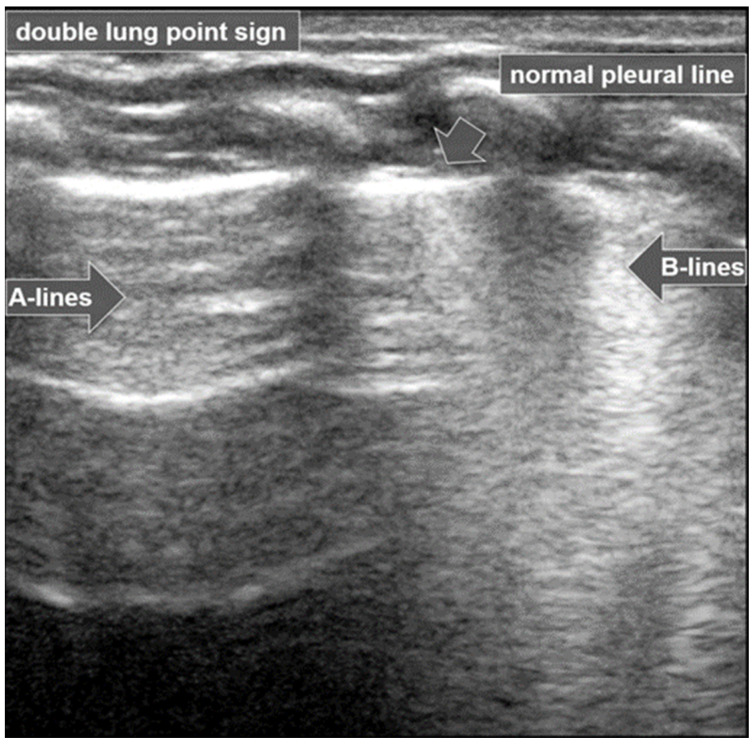
Double-lung-point sign in Transient Tachypnea of the Newborn.

The hallmark LUS finding of TTN is the double-lung-point (Figure 11), a clear demarcation between the inferior lung fields, which appear normally aerated, and the superior fields, which show dense, confluent B-lines consistent with interstitial fluid accumulation. As previously mentioned, the double-lung-point has been reported with a specificity as high as 100% for TTN [6,17,26,36]. Notably, this LUS pattern is not typically observed in other neonatal pulmonary conditions such as pneumonia, pneumothorax, atelectasis, or pulmonary hemorrhage. The presence of these features in LUS, particularly the double-lung point and the absence of subpleural consolidations, reinforces the diagnostic value of point-of-care lung ultrasound in distinguishing TTN from other causes of neonatal respiratory distress [20,29,37,38,39].

#### 3.2.4. Respiratory Distress Syndrome (RDS)

**Figure 12:**
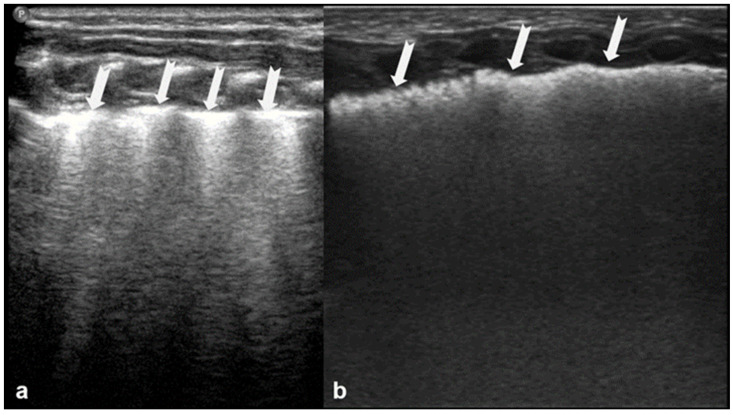
A frequent and characteristic imaging finding in RDS is the presence of B-Lines, bilaterally and throughout the lung parenchyma, in this case bilateral B-lines with no normal parenchyma (**a**) (absence of A-lines). Also, note the abnormal thickening ((**a**,**b**) arrows) of the pleural line (**b**). The overall sense is that of white lung.

**Figure 13:**
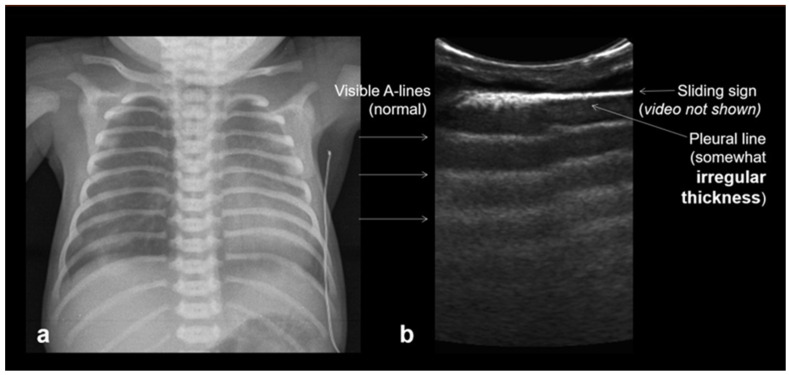
Normal to mild RDS: by chest X-ray (**a**); by LUS (**b**).

**Figure 14:**
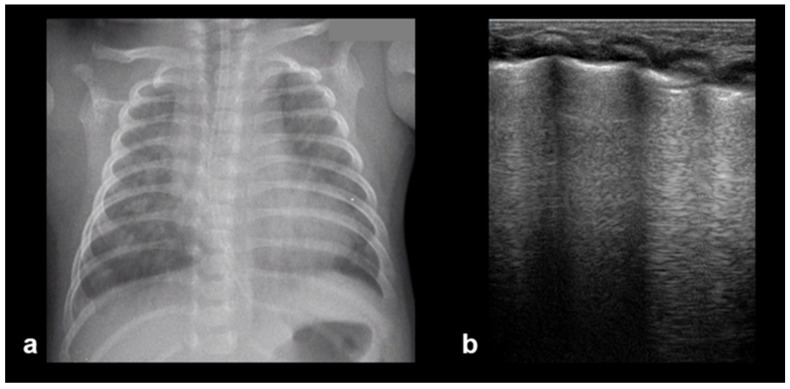
Severe RDS shown on chest X-ray (**a**); LUS (**b**). The ability of the LUS to characterize the severity of the RDS is debatable.

**Figure 15:**
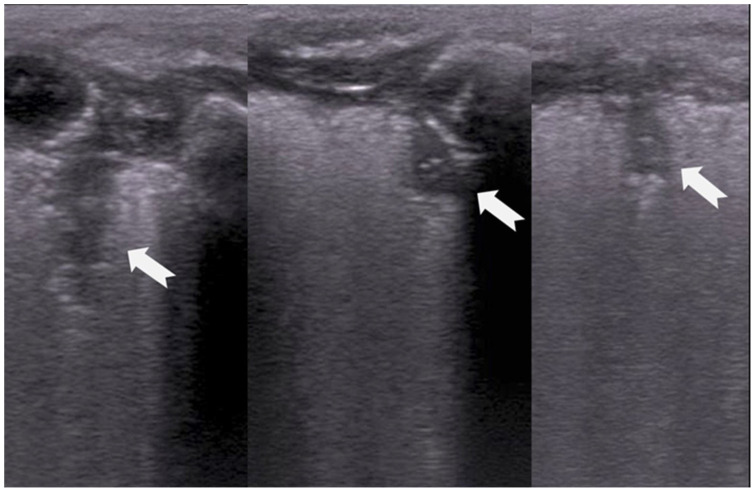
Severe RDS with sub-segmental(-pleural) consolidations. by LUS.

In RDS, lung ultrasound findings include consolidation or bilateral “white lung”, aero bronchograms, pleural line abnormalities, and the absence of A-lines (Figure 12, Figure 13, Figure 14 and Figure 15) [30,35,40,41].

In RDS, specific LUS patterns describing lung aeration accurately predict the need for surfactant administration [35].

**Figure 16:**
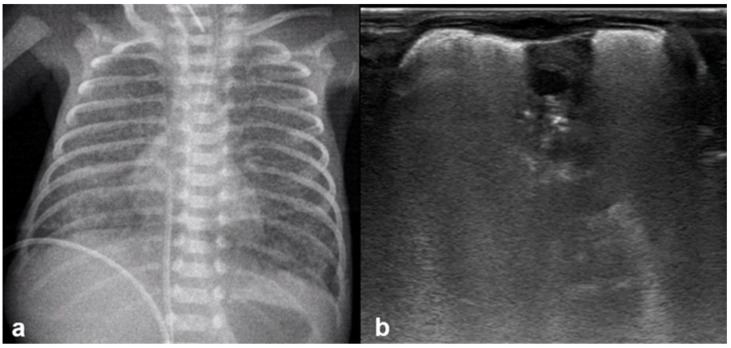
Interstitial emphysema shown on chest x-ray (**a**) and LUS (**b**). This case underlines the superiority of CXR in differentiating this pathology from RDS, which has a similar LUS appearance.

RDS is a common cause of neonatal respiratory failure, particularly in preterm infants, resulting from surfactant deficiency and alveolar collapse. LUS has become a valuable diagnostic tool for RDS, offering real-time visualization of lung pathology at the bedside [38,42,43,44,45]. A hallmark sonographic feature of RDS is the presence of dense, confluent B-lines throughout the lung fields, producing a “white lung” appearance due to the loss of normal aeration. Notably, the whole lung is typically involved, with a diffuse, bilateral distribution that distinguishes RDS from more localized pulmonary conditions [37,46,47]. Additionally, interspersed consolidations may be present, including hypoechoic and subpleural areas of alveolar collapse, reflecting regions of complete de-aeration. These consolidations often lack air bronchograms and are associated with irregular or thickened pleural lines. The combination of diffuse B-line patterns, global lung involvement, and patchy consolidations provides a highly suggestive ultrasound profile of RDS and facilitates early diagnosis. In addition, for RDS, LUS may be used for severity assessment and the monitoring of treatment response in the neonatal intensive care setting [16,20,27,29]. The ability of the LUS to characterize the severity of the RDS is debatable (Figure 14 and Figure 16) [48].

#### 3.2.5. Meconium Aspiration Syndrome (MAS)

**Figure 17:**
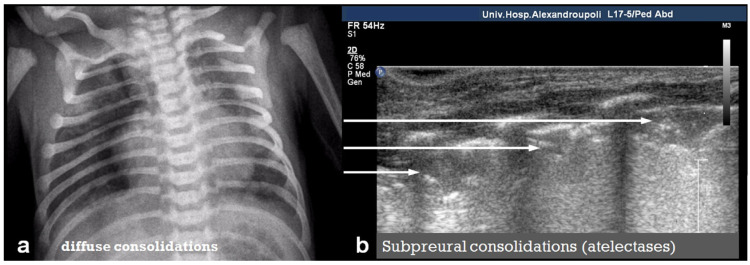
Meconium aspiration syndrome: (**a**) CXR with small diffuse consolidations, (**b**) LUS reveals coalescent B-lines and small subpleural lung consolidations (arrows).

MAS is a serious respiratory condition that occurs when a newborn infant inhales meconium-stained amniotic fluid, leading to airway obstruction, chemical pneumonitis, and impaired gas exchange. LUS in MAS typically reveals diffuse lung consolidations, often bilateral and patchy, reflecting widespread alveolar involvement due to inflammation, atelectasis, or surfactant inactivation (Figure 17).

These consolidations may appear as subpleural hypoechoic areas with hepatization and air bronchograms [37]. In addition, both focal and confluent B-lines may be observed, indicating interstitial fluid accumulation secondary to inflammation or pulmonary edema. Pleural irregularities, including thickening and disruption of the pleural line, are also common and suggest subpleural injury or inflammation. The combination of these findings in conjunction with a perinatal clinical history can help to differentiate MAS from other neonatal respiratory disorders such as Transient Tachypnoea of the Newborn (TTN) or Respiratory Distress Syndrome (RDS) and supports the use of LUS as a valuable bedside diagnostic tool in the neonatal intensive care setting [8,16,17,29,49,50].

#### 3.2.6. Bronchopulmonary Dysplasia (BPD)

**Figure 18:**
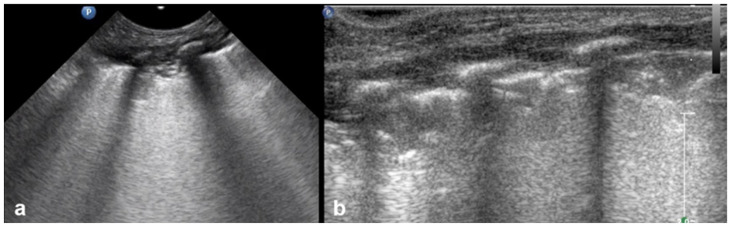
Bronchopulmonary dysplasia: heterogeneous pulmonary involvement with “solid” and coalescent B-lines (**a**), with an additional thickened and irregular pleural line (**a**,**b**). The US pattern described seems to be wholly inadequate and not useful from a diagnostic point of view.

**Figure 19:**
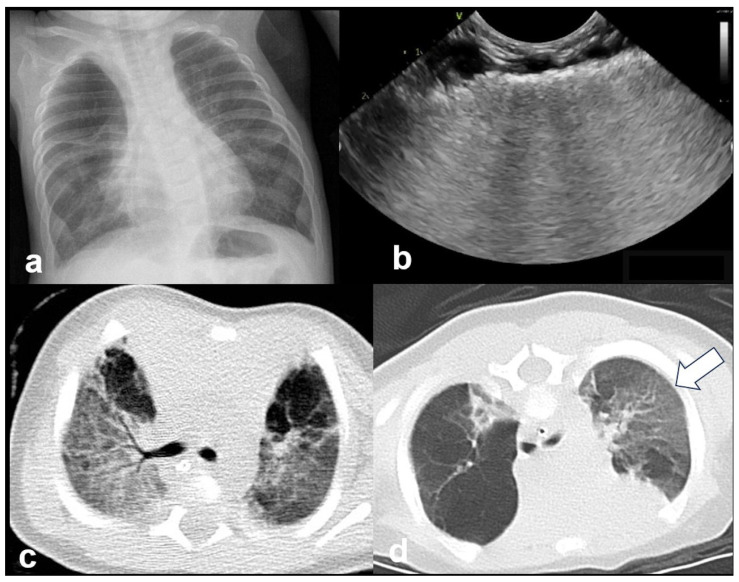
Bronchopulmonary dysplasia: (**a**) CXR; (**b**) LUS: abnormal thickening of pleural line; (**c**,**d**) CT. The main findings from all methods include lung parenchymal changes unequally distributed and contiguous to spared areas (arrow in (**d**)).

BPD is a chronic lung disease primarily affecting preterm infants who required prolonged mechanical ventilation or oxygen therapy. LUS findings in BPD often reveal an abnormal distribution of aeration patterns, reflecting heterogeneous pulmonary involvement. One of the hallmark features of BPD is the presence of areas with “solid” or coalescent B-lines, indicating interstitial fibrosis or chronic lung remodeling. In addition, the pleural line may appear to be abnormally thickened and irregular, which is consistent with chronic inflammation and structural alterations to the lung periphery. Subpleural thickenings of varying sizes can also be seen scattered throughout the lung fields, representing localized fibrosis or areas of reduced aeration (Figure 18 and Figure 19).

Taken together, these findings, coupled with a history of prolonged mechanical ventilation, provide a non-invasive method of assessing the severity and progression of BPD in neonates and can assist in differentiating it from acute respiratory conditions [16,20,47].

#### 3.2.7. Lung Consolidation

**Figure 20:**
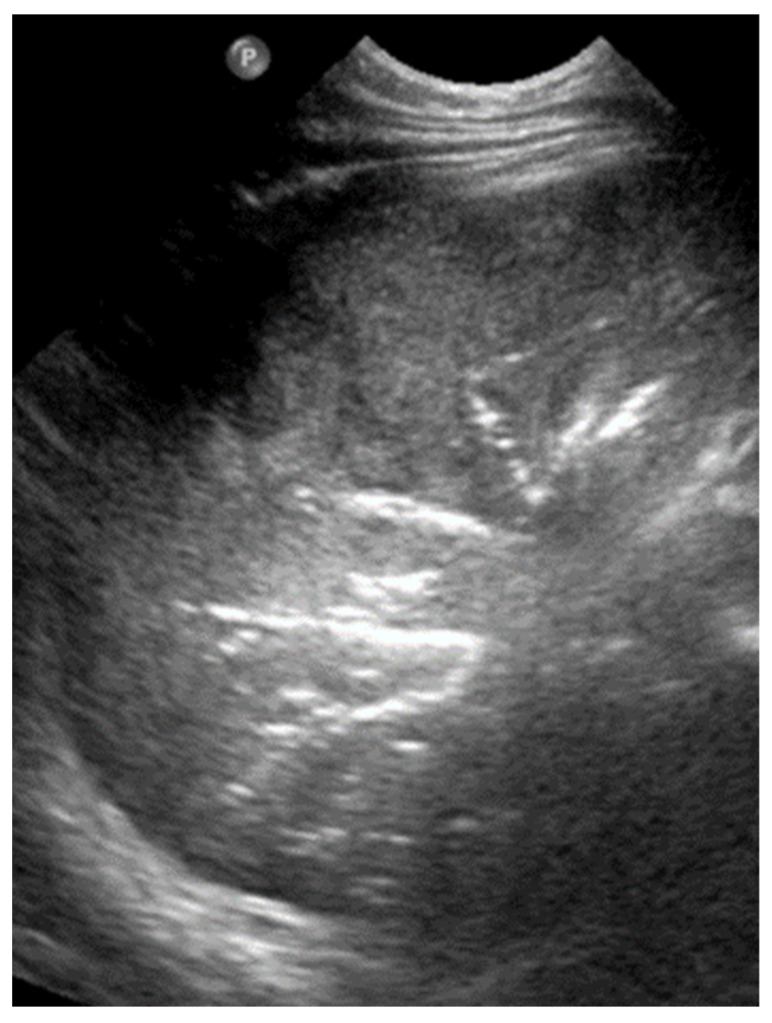
Lung consolidation (alveolar filling with exudate/pus): hepatization of lung parenchyma containing air bronchogram (normal branching pattern of the air-filled bronchi).

In LUS, pulmonary consolidation refers to the replacement of normally aerated lung tissue with fluid, cells, or other pathological material, leading to a loss of the usual air-filled ultrasound pattern. The characteristic LUS signs of alveolar pathology, such as pneumonia, include the absence of the pleural line, the presence of air bronchograms, and hepatization of the lung parenchyma due to alveolar filling with exudates or pus (Figure 20).

**Figure 21:**
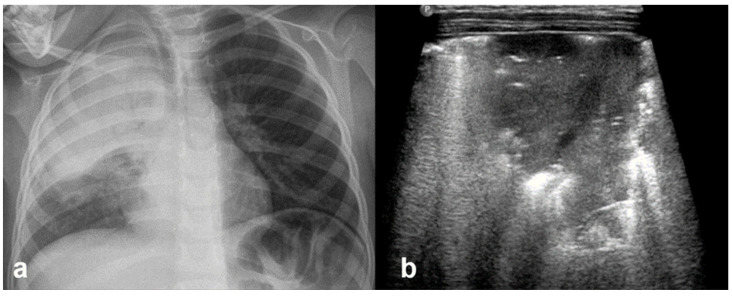
Shred sign: (**a**) CXR: lung consolidation in right upper lobe; (**b**) LUS: area with irregular margins and heterogeneous echogenicity.

Pneumonia is also identified by the appearance of an irregular echogenic artifact, known as the “shred sign”, which is created by the inhomogeneous interface between consolidated (non-ventilated) and aerated lung tissue (Figure 21) [12,13,20,26,51].

One of the most distinctive sonographic features of consolidation is the hepatization of the subpleural lung tissue, where the affected area appears with an echotexture similar to liver tissue. This occurs due to the dense accumulation of inflammatory exudates within the alveoli, causing the lung to become uniformly solid in appearance. The consolidated lung often extends to the pleural surface and may exhibit associated signs, such as dynamic air bronchograms. The presence of a dynamic air bronchogram in LUS is a sign that helps distinguish pneumonia from other conditions such as atelectasis. It refers to air-filled bronchioles (airways) that move synchronously with respiration, indicating that they are open and connected to larger airways [31,52]. This movement contrasts static air bronchograms, which remain fixed, and suggests that the airways are obstructed. In addition, it presents with other signs, such as irregular pleural lines and pleural effusion. In neonates, lung consolidation is most seen in cases of pneumonia, atelectasis, or severe respiratory distress syndrome, and its identification is crucial for guiding appropriate management and follow-up care [12,20].

#### 3.2.8. Pneumothorax

LUS is a highly sensitive and specific modality for the rapid bedside diagnosis of pneumothorax, particularly in critical care and neonatal settings.

**Figure 22:**
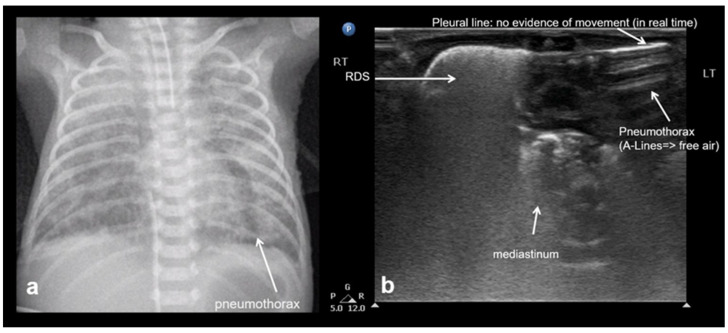
Pneumothorax: (**a**) CXR, (**b**) LUS.

The hallmark sonographic feature of pneumothorax is the absence of lung sliding, reflecting a separation between the parietal and visceral pleura due to the presence of intrapleural air. This absence is typically accompanied by the loss of B-lines, which are reverberation artifacts dependent on intact pleural contact and subpleural aeration. Instead, A-lines are observed, indicating the presence of air in the pleural space without lung parenchymal motion. When pneumothorax is evaluated in M-mode, a characteristic stratosphere sign, or “barcode sign”, replaces the normal “sea-shore sign”, showing uniform horizontal lines without the granular pattern associated with moving lung tissue. The combination of the absence of lung sliding, the absence of B-lines, the presence of prominent A-lines, and the presence of the stratosphere sign strongly supports the diagnosis of pneumothorax and helps differentiate it from other causes of respiratory distress (Figure 22) [12,13,15,20,28,29,40,53].

#### 3.2.9. Pneumomediastinum

The presence of large A-line artifacts or small echogenic lines at the lateral borders of the thymus, or in its parenchyma in the suprasternal plane, strongly suggests pneumomediastinum. However, it should be noted that the LUS characteristics of pneumomediastinum have not yet been defined [12,18].

#### 3.2.10. Pleural Effusion

Pleural effusion, defined as the accumulation of fluid in the pleural space, can be easily detected by LUS, which is highly sensitive and able to identify even small volumes of fluid (as little as 5–20 mL). In contrast, effusions greater than 175–250 mL must typically be present for the detection of pleural effusion by conventional chest X-rays, although lateral decubitus views may detect effusions of approximately 50 mL. A pure pleural effusion typically appears as an anechoic collection located between the parietal and visceral pleura, often seen as a clear, dark space (fluid) that allows for the visualization of underlying lung structures and diaphragm movement [13,20,35,54].

In contrast, complicated effusions may exhibit internal echogenicity due to the presence of fibrin, debris, or infection. Identifying the nature of the effusion, in other words whether it is simple effusion or complicated, is critical for clinical decision-making, and may help indicate the need for drainage, antibiotic therapy, or surgical intervention. Thus, LUS can be considered to play a pivotal role in both the diagnosis and management of pleural effusions, particularly in neonates and critically ill patients [10,23].

#### 3.2.11. Empyema

Empyema refers to the accumulation of purulent material within the pleural space, typically as a complication of pneumonia or other infectious processes involving the lungs. In neonates and pediatric patients, empyema is a significant cause of morbidity and often presents with respiratory distress, fever, and signs of pleural effusion. LUS is a valuable tool for the evaluation of empyema, because it often offers greater sensitivity than chest X-rays and can be used to effectively guide both diagnosis and management [6,12,54].

LUS findings in empyema typically reveal a complex pleural effusion rather than a simple anechoic collection. The effusion often contains internal echogenic debris, fibrinous strands, and multiple septations that create a multiloculated appearance. These features help differentiate empyema from uncomplicated effusions. In some cases, the adjacent lung may appear consolidated, and pleural thickening is commonly observed. The consolidated lung adjacent to the empyema may show reduced or absent air bronchograms, reflecting impaired ventilation due to compression or infection.

**Figure 23:**
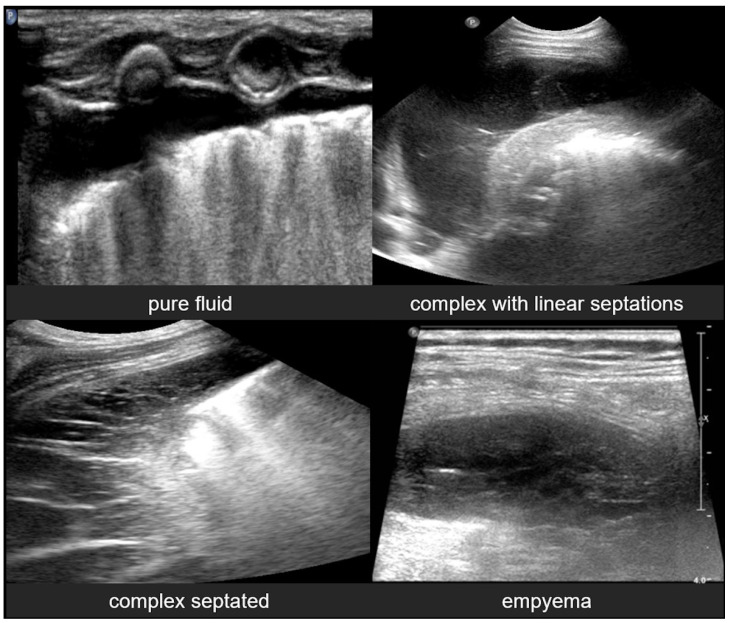
Pleural effusions.

Timely recognition of pleural empyema is critical, because it requires aggressive intervention, including antibiotics and often image-guided or surgical drainage. Thus, lung ultrasound not only facilitates early detection of pleural empyema, but also plays a crucial role in procedural planning, monitoring therapeutic response, and reducing reliance on ionizing radiation in vulnerable pediatric populations (Figure 23) [6,12].

## 4. Future Directions

The everyday use of LUS in clinical practice remains a subject of ongoing observation and research across multiple areas. One such area is the investigation of the position of long line catheter tips and the placement of the endotracheal tube.

LUS has also been applied to the assessment of the diaphragm and airways, particularly in the real-time guidance of endotracheal tube placement, confirmation of its correct positioning, and monitoring during the extubation process. Furthermore, so called “virtual spirometry” has emerged as a promising method, based on measurements of diaphragmatic muscle in inspiration vs. expiration.

Further studies are needed to evaluate the clinical value and diagnostic accuracy of LUS in a variety of clinical situations.

## 5. Conclusions

LUS is increasingly recognized as a reliable, standard method for the diagnosis of respiratory diseases in the pediatric population, including newborn infants, such as hyaline membrane disease, Transient Tachypnea of the Newborn, and meconium aspiration syndrome. It can also play a key role in guiding surfactant therapy and predicting clinical outcomes. LUS can be performed at the bedside in both emergency departments and neonatal intensive care units, making it a highly accessible method for evaluation and monitoring of critically ill neonates.

One of LUS’s major advantages is that it allows for a significant reduction in radiation exposure compared to chest X-rays. While LUS does not completely replace chest X-rays, it significantly decreases their necessity in clinical practice, offering clear benefits, including minimization of irradiation risks.

## Data Availability

The datasets generated during and/or analyzed during the current study are available from the corresponding author on reasonable request.

## Abbreviations

The following abbreviations are used in this manuscript:

LUS	Lung Ultrasound
TTN	Transient Tachypnoea of the Newborn
RDS	Respiratory Distress Syndrome
AIS	Alveolar–Interstitial Syndrome
BPD	Bronchopulmonary Dysplasia
MAS	Meconium Aspiration Syndrome
